# Endoscopic management of pancreatic duct injury by endoscopic stent placement: a case report and literature review

**DOI:** 10.1186/1749-7922-7-21

**Published:** 2012-07-12

**Authors:** Yasuhiro Ito, Takeshi Kenmochi, Tomoyuki Irino, Tomohisa Egawa, Shinobu Hayashi, Atsushi Nagashima, Nao Hiroe, Mitsuhide Kitano, Yuko Kitagawa

**Affiliations:** 1 Department of Surgery, Saiseikai Yokohamashi Tobu Hospital, 3-6-1, Shimosueyoshi, Tsurumi-ku, Yokohama-shi, Kanagawa, 230-8765, Japan; 2 Department of Trauma and Emergency Surgery, Saiseikai Yokohamashi Tobu Hospital, 3-6-1, Shimosueyoshi, Tsurumi-ku, Yokohama-shi, Kanagawa, 230-8765, Japan; 3 Department of Surgery, Keio University School of Medicine, 35 Shinanomachi, Shinjuku-ku, Tokyo, 160-8582, Japan

**Keywords:** Pancreatic injury, Endoscopic, Pancreatic stent, Endoscopic nasopancreatic drainage

## Abstract

Recently, the diagnostic evaluation of pancreatic injury has improved dramatically. On the other hand, it is occasionally difficult to diagnose pancreatic injury, because there are no specific signs, symptoms, or laboratory findings. Radiological imaging also often fails to identify pancreatic injury in the acute phase. Delayed diagnosis results in significant morbidity and mortality. Most cases of pancreatic injury with suspicion or pancreatic duct disruption require surgery. Endoscopic retrograde cholangiopancreatography is one of the most accurate modalities for ductal evaluation and therapy and might enable one to avoid unnecessary surgery. We describe endoscopic management of pancreatic duct injury by endoscopic stent placement. A 45-year-old woman was admitted after a traffic accident. A computed tomography scan showed pancreatic parenchyma disruption at the pancreatic head. Endoscopic retrograde cholangiopancreatography demonstrated disruption of the pancreatic duct with extravasation into the peripancreatic fluid collection. A 5-French endoscopic nasopancreatic drainage (ENPD) tube was placed. Her symptoms dramatically improved. ENPD tube was exchanged for a 5-French 5-cm pancreatic stent. Subsequent follow-up CT revealed remarkable improvement. On the 26th day, the patient was discharged from the hospital without symptoms or complications. In this report, a pancreatic stent may lead to rapid clinical improvement and enable surgery to be avoided. On the other hand, the reported complications of long-term follow-up make the role of stenting uncertain. Thus, close attention should be paid to stenting management in the follow-up period. A pancreatic stent is useful for pancreatic ductal injury. If pancreati*c* ductal injury is managed appropriately, a pancreatic stent may improve the clinical condition, and also prevent unnecessary surgery.

## Introduction

Pancreatic injury is uncommon, because the retroperitoneal location of the pancreas offers relative protection. In addition, the clinical presentation is often subtle, frequently resulting in delayed treatment. Radiological imaging often fails to identify pancreatic injury in the acute phase. Delayed diagnosis results in significant morbidity and mortality. Thus, diagnosis must be managed strictly. Although conservative treatment for minor pancreatic injury is widely accepted, the treatment of pancreatic duct injury is still controversial. Most cases of pancreatic injury with suspicion or evidence of pancreatic duct disruption require surgery, even if there is suspected pancreatic duct injury. Endoscopic retrograde cholangiopancreatography (ERCP) is one of the most accurate modalities for ductal evaluation and therapeutic management. If the patient is awake and alert with stable vital signs, ERCP might enable one to avoid unnecessary surgery.

In this study, we report a case of endoscopic management of pancreatic duct injury by endoscopic stent placement.

## Case presentation

**Figure 1:**
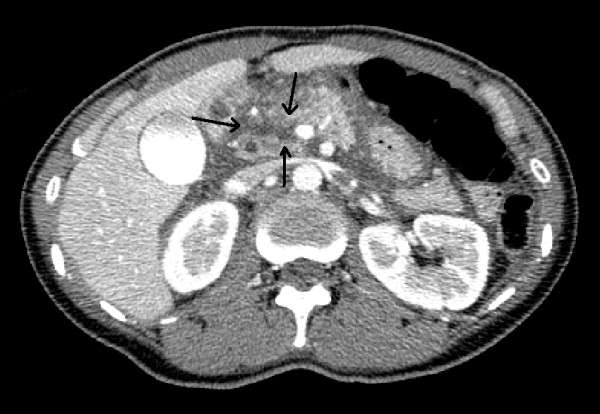
A computed tomography scan showed pancreatic parenchyma disruption with a small amount of peripancreatic fluid at the pancreatic head.

**Figure 2:**
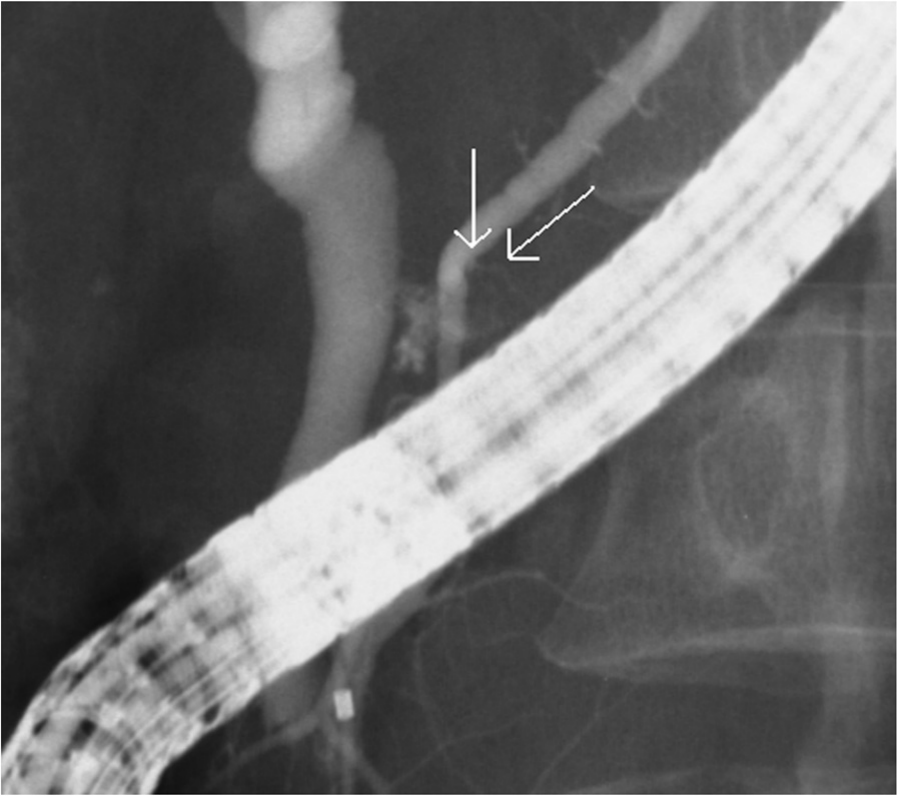
**Endoscopic retrograde cholangiopancreatography demonstrated disruption of the pancreatic duct with extravasation into the peripancreatic fluid collection (arrow)**.

**Figure 3:**
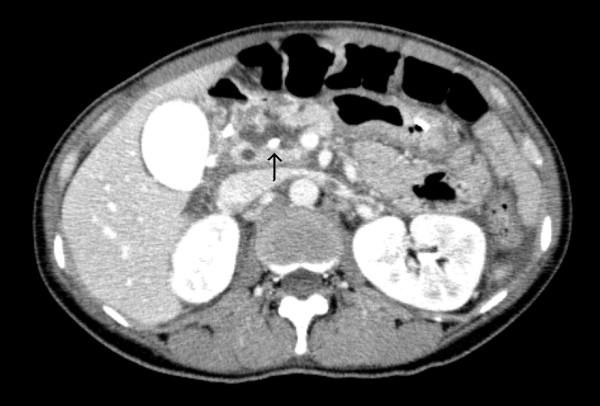
**A computed tomography scan after endoscopic retrograde cholangiopancreatography revealed endoscopic nasopancreatic drainage tube (arrow) placed into pancreatic duct, and there was no exacerbation of pancreatic injury or fluid collection**.

**Figure 4:**
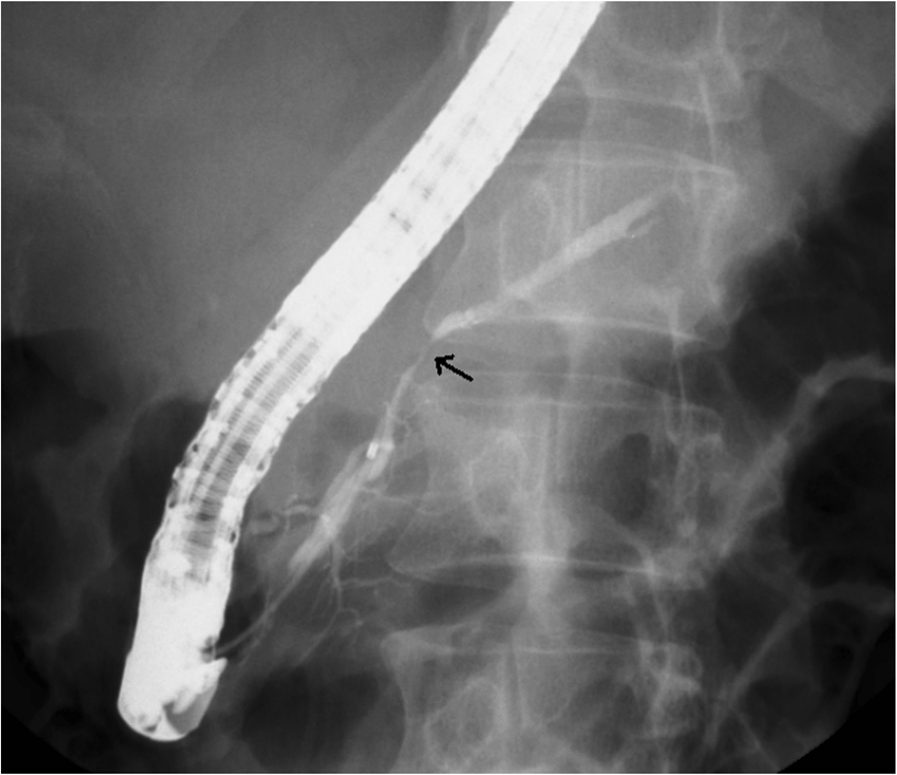
**Endoscopic retrograde cholangiopancreatography revealed a mild stricture (arrow) at the injured duct without leakage**.

**Figure 5:**
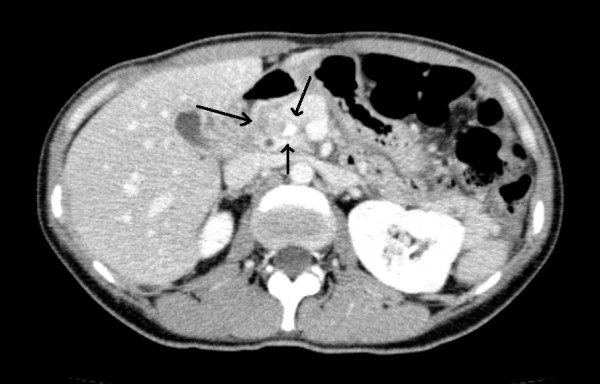
**A computed tomography scan after tube exchange revealed remarkable improvement of the injured pancreatic parenchyma and resolution of the peripancreatic fluid collection**.

A 45 year old woman was a seat-belted driver in a motor vehicle. She was admitted to a local hospital after a traffic accident. The patient was awake and alert with stable vital signs and was complaining of abdominal pain. An urgent computed tomography (CT) scan showed pancreatic parenchyma disruption with a small amount of peripancreatic fluid at the pancreatic head (Figures 1). The patient was transferred to our hospital for further management 40 hours after the traffic accident. When the patient was admitted to our hospital, her vital signs were normal. Laboratory examinations revealed a white blood cell (WBC) count 14400/μL (normal 3500–8500), serum amylase (AMY) 1321 IU/L (normal 40–126), and C-reactive protein (CRP) 6.8 mg/dL (normal 0.0-0.5). Endoscopic retrograde cholangiopancreatography (ERCP) demonstrated disruption of the pancreatic duct with extravasation into the peripancreatic fluid collection (Figures 2). A 5-French endoscopic nasopancreatic drainage (ENPD) tube was placed into the pancreatic duct across the duct disruption. A CT scan after ERCP revealed ENPD tube placed into pancreatic duct, and there was no exacerbation of pancreatic injury or fluid collection (Figures 3). Her symptoms dramatically improved upon endoscopic treatment. ERCP on the 17th day after admission revealed a mild stricture at the injured duct without leakage (Figures 4), and the ENPD tube was exchanged for a 5-French 5-cm endoscopic pancreatic stent (EPS). Subsequent follow-up CT after tube exchange revealed remarkable improvement of the injured pancreatic parenchyma and there is no fluid collection at the pancreatic head (Figures 5). On the 26th day, the patient was discharged from the hospital without symptoms or complications. Amylase remained within the normal range after ENPD drainage. Routine laboratory examinations were normal and EPS remain in situ.

## Discussion

Pancreatic injury occurs in only 3% to 12% of all patients with severe abdominal trauma [1]. The morbidity and mortality rates of pancreatic injury are high [2,3]. Many pancreatic injuries remain undetected at first, and only become apparent when complications arise or other injuries are present; in more than 80% of patients, at least one other abdominal organ is also injured [4]. Recently, the diagnostic evaluation of pancreatic injury has improved dramatically [5]. On the other hand, it is occasionally difficult to diagnose pancreatic injury, because there are no specific signs, symptoms, or laboratory findings. Therefore, proper diagnosis and treatment of pancreatic injury in the acute phase is indispensable. Delays in diagnosis and inappropriate treatment, in part, lead to the significant complications of pancreatic injury, including pseudocysts, fistulae, chronic pancreatitis, abscess formation, and sepsis.

Magnetic resonance cholangiopancreatograpy (MRCP) is a non-invasive diagnostic tool which may enable the detection of pancreatic duct injury. The use of MRCP is recommended in hemodynamically stable patients [6] and it also allows detection of specific pancreas-related complications [7]. On the other hands, the advantage of MRCP is reported that MRCP does not provide real-time visualization of ductal filling and extravasation. For this reason, MRCP does not allow for confirmation of ductal communication with a pancreatic pseudocyst or other fluid collection [6].

Gougeon et al. reported a diagnostic approach to pancreatic injury by ERCP in 1976[8]. Although it is invasive, ERCP is the most accurate diagnostic tool for ductal evaluation, and it can also be used to provide treatment. However, delays in ERCP have led to significantly higher complication rates. Early ERCP was found to be associated with significantly fewer pancreas-related complications than later ERCP [9]. Although ERCP is the most useful procedure for the diagnosis of pancreatic ductal injury in stable patients, surgery should be considered without hesitation if the patient’s condition is unstable.

Most pancreatic injuries involving hematomas and small tears without pancreatic ductal disruption are generally managed conservatively with observation and selective drainage. In contrast, injuries of grade III and IV, according to the pancreatic organ injury scale of the American Association for the Surgery of Trauma (AAST) (Table 1) [10], are controversial. Since many authors argue in favor of an early operative intervention to prevent increased morbidity caused by delay, they recommend surgery and the surgical removal of the organ when the duct is involved [3]*.* There are a number of alternative procedures that can be used for the management of grade IV injury, such as duodenal diversion, pyloric exclusion, the Whipple procedure, or simple drainage, with the choice dependent on the patient’s hemodynamic status and the presence or absence of associated duodenal injury [11,12]. Sometimes, the decision to do a pancreaticoduodenectomy is unavoidable. If patient is hemodynamically unstable, it should be performed as a two-step procedure. After the initial damage control surgery, anastomoses are completed at a second surgery when the patient is stable.

**Table 1:** Classification of pancreatic trauma (AAST)

**Grading**	**Injury**	**Description**
Grade I	Hematoma	Mild contusion without duct injury
	Laceration	Superficial laceration without duct injury
Grade II	Hematoma	Major contusion without duct injury
	Laceration	Major laceration without duct injury or tissue loss
Grade III	Laceration	Distal transection or parenchymal injury with duct injury
Grade IV	Laceration	Proximal transection or parenchymal injury involving the ampulla
Grade V	Laceration	Massive disruption of the pancreatic head

Initial management of pancreatic injury is the accurate definition of the degree of pancreatic injury using a CT and MRCP. ERCP has been until recently the most accurate method for detecting pancreatic duct injury in hemodynamically stable patients. Then, the pancreatic stent is placed into the pancreatic duct across the duct disruption if there is evidence of pancreatic injury from pancreatography. Unfortunately, when patients are hemodynamically unstable or complaining of persistent abdominal pain despite the proper management, it should not hesitate to surgery.

Recently, some case series have shown pancreatic duct stent placement to be an effective therapy in resolving pancreatic duct disruption (Table 2) [9,13-25]. Although stent therapy can improve the clinical condition and resolve fistula and pseudocyst, ductal stricture is a major complication in the long term. Ductal changes can be caused by the trauma itself or they may be induced by the pancreatic stent, resulting either from stent occlusion and direct stent trauma or from side-branch occlusion. Ikenberry et al. reported the longer stent placement had a higher stent-occlusion rate and an increased risk of ductal stricture [26]. In the pancreatic head, 7 cm is enough, and 9, 12, or 15 cm can be used for the body and tail. We place the stent across the disruption when possible. Although we avoid surgical management, stent exchanges may be required because of long-term complications, including pancreatic ductal stricture. Lin et al. reported that the average times for stent exchange and duration of stenting in patients with severe ductal stricture were 8 times and 25 months, respectively [16]. The diameter of the major pancreatic duct is the main factor in ductal stricture. The normal diameter of the major pancreatic duct varies from 2 to 3 mm in the body and 3 to 4 mm in the head, and the healing process in the injured duct makes stricture impossible to avoid, even with stent placement. After a ductal stricture forms, it is treated with repeated stenting. Another factor in stricture is the severity of ductal injury. The period of stent placement is not sufficiently clear at this time. Long-term follow-up has shown that complications resulting in ductal stricture make the role of pancreatic stents uncertain. In addition, complications caused by a stent are rare but have been described, including occlusion, migration, duodenal erosion, and infection [27]*.* Pancreatic stent placement is not risk free. A case of sepsis that developed after stenting was reported, and the patient died [16]. Chronic renal failure may be a risk factor, and contrast medium leaking into the retroperitoneal space is another. When contrast medium leaks into the retroperitoneal space or even into the peritoneal cavity, the injury is more serious, and surgery is suggested [28]*.* Therefore, the process for treatment of pancreatic injury must be managed prudently.

**Table 2:** Reported cases of pancreatic duct injury with an endoscopic stent

**Authors**	**Age**	**Gender**	**Mechanism**	**Location**	**Treatment**	**Outcome**	**Reference**
Cattaneo SM et al.	17	F	Blunt	body - tail	Pancreatic stent, no operation	Nothing	[13]
Canty TG Sr et al.	9	F	Blunt	body	Pancreatic stent, no operation	Mild stricture	[14]
	8	M	Blunt	tail	Pancreatic stent, no operation	Nothing	
Wolf A et al.	24	F	Blunt	head - body	Pancreatic stent, no operation	Nothing	[15]
Lin BC et al.	37	F	Blunt	head	Surgical drainage → Pancreatic stent	Migration	[16]
	36	M	Blunt	body - tail	Surgical drainage → Pancreatic stent	Severe stricture	
	61	F	Blunt	body	Pancreatic stent → Distai pancreatectomy	Death	
	18	M	Blunt	body	Pancreatic stent, no operation	Severe stricture	
	28	M	Blunt	head	Pancreatic stent, no operation	Mild stricture	
Huckfeldt R et al.	27	F	Blunt	head	Pancreatic stent, no operation	Nothing	[17]
Abe T et al.	43	M	Blunt	head	Pancreatic stent, no operation	Mild stricture	[18]
Bagci S et al.	21	M	Blunt	body	Pancreatic stent, no operation	Mild stricture	[19]
Cay A et al.	11	M	Blunt	body	Pancreatic stent, no operation	Nothing	[20]
Hsieh CH et al.	36	M	Blunt	head, body (2sites)	Pancreatic stent, no operation	Slight excavation	[21]
Hashimoto A et al.	60	M	Blunt	head	Pancreatic stent, no operation	Nothing	[22]
Houben CH et al.	11	M	Blunt	head (neck)	Pancreatic stent → Cyst-gastrostomy	not described	[23]
	11	F	Blunt	body	Pancreatic stent → Cyst-gastrostomy	not described	
	9	M	Blunt	head (neck)	Pancreatic stent, no operation	not described	
Bendahan J et al.	22	M	Penetrating	head	Surgical drainage → Pancreatic stent	Nothing	[24]
Rastogi M et al.	28	M	Penetrating	head	Surgical drainage → Pancreatic stent	Nothing	[25]
Kim HS et al.	46	M	not described	head	Pancreatic stent, no operation	Mild stricture in 2 of 3 patients	[9]
	35	M	not described	pancreas fracture	Pancreatic stent, no operation		
	40	F	not described	body	Pancreatic stent, no operation		

In our case, CT revealed disruption of the pancreatic parenchyma at the time of admission. Fortunately the patient’s hemodynamic status was stable, and we could successfully perform the endoscopic procedure. We considered that the ENPD tube was correctly placed to drain the pancreatic juice and to avoid stent migration, dropping out, and occlusion. Although the patient could avoid more invasive surgery in the acute phase, she developed the complication of pancreatic stricture as a result of the healing process. This procedure may lead to rapid clinical improvement and enable surgery to be avoided. On the other hand, the reported complications of long-term follow-up make the role of stenting uncertain. Thus, close attention should be paid to stenting management in the follow-up period.

## Conclusion

Pancreatic stent is useful for pancreatic ductal injury. If the indication, timing, and patient condition for pancreatic ductal injury are managed appropriately, placement of a pancreatic stent can improve the clinical condition, and it may also prevent unnecessary surgery. Since problems with pancreatic stent remain, further investigation is needed.

## Consent

Written informed consent was obtained from the patient for publication of this case report and accompanying images. A copy of the written consent is available for review by the Editor-in-Chief of this journal.

## Competing interests

The authors declare that they have no ethical or completing interests.

## Authors’ contributions

All authors contributed to researching, editing and writing the article. All authors read and approved the final manuscript.
